# Indicators to evaluate organisational knowledge brokers: a scoping review

**DOI:** 10.1186/s12961-020-00607-8

**Published:** 2020-08-24

**Authors:** Julia Scarlett, Birger C. Forsberg, Olivia Biermann, Tanja Kuchenmüller, Ziad El-Khatib

**Affiliations:** 1 Department of Global Public Health, Karolinska Institutet, Tomtebodavägen 18a, SE-171 77 Stockholm, Sweden; 2 Region Stockholm, Hantverkargatan 11B, 112 21 Stockholm, Sweden; 3 World Health Organization Regional Office for Europe, UN City, Marmorvej 51, DK-2100 Copenhagen Ø, Denmark; 4 World Health Programme, Université du Québec en Abitibi-Témiscamingue (UQAT), 445 Boulevard de l’Université, Rouyn-Noranda, QC J9X 5E4 Canada

**Keywords:** Knowledge translation, Policy-making, Capacity-building, Programme evaluation, Indicators

## Abstract

**Background:**

Knowledge translation (KT) is currently endorsed by global health policy actors as a means to improve outcomes by institutionalising evidence-informed policy-making. Organisational knowledge brokers, comprised of researchers, policy-makers and other stakeholders, are increasingly being used to undertake and promote KT at all levels of health policy-making, though few resources exist to guide the evaluation of these efforts. Using a scoping review methodology, we identified, synthesised and assessed indicators that have been used to evaluate KT infrastructure and capacity-building activities in a health policy context in order to inform the evaluation of organisational knowledge brokers.

**Methods:**

A scoping review methodology was used. This included the search of Medline, Global Health and the WHO Library databases for studies regarding the evaluation of KT infrastructure and capacity-building activities between health research and policy, published in English from 2005 to 2016. Data on study characteristics, outputs and outcomes measured, related indicators, mode of verification, duration and/or frequency of collection, indicator methods, KT model, and targeted capacity level were extracted and charted for analysis.

**Results:**

A total of 1073 unique articles were obtained and 176 articles were qualified to be screened in full-text; 32 articles were included in the analysis. Of a total 213 indicators extracted, we identified 174 (174/213; 81.7%) indicators to evaluate the KT infrastructure and capacity-building that have been developed using methods beyond expert opinion. Four validated instruments were identified. The 174 indicators are presented in 8 domains based on an adaptation of the domains of the Lavis et al. framework of linking research to action – general climate, production of research, push efforts, pull efforts, exchange efforts, integrated efforts, evaluation and capacity-building.

**Conclusion:**

This review presents a total of 174 method-based indicators to evaluate KT infrastructure and capacity-building. The presented indicators can be used or adapted globally by organisational knowledge brokers and other stakeholders in their monitoring and evaluation work.

## Background

Health research is largely an untapped resource in policy-making; while there is an abundance of health research conducted worldwide, the translation of research into policy can be slow or even non-existent [1, 2]. Knowledge translation (KT) — a dynamic and iterative process that includes synthesis, dissemination, exchange and ethically sound application of knowledge [3] — has been advocated in high level policy meetings and echoed in practice by major health policy actors like WHO as a key approach for linking research and policy [4–7]. KT has been implemented in a variety of disciplines using several different, often overlapping frameworks [8–13]. The Lavis et al. [14] framework developed to assess country-level efforts to link research to action has been widely adopted in the health policy arena [14–17]. The framework provides four key domains to guide country-level KT efforts, namely (1) the climate for research use; (2) the production of research that is both highly relevant to and appropriately synthesised for research users; (3) the mix of clusters of activities used to link research to action; and (4) the evaluation of efforts to link research to action [14]. Within their framework, Lavis et al. classify KT activities into four models of push, pull, exchange and integrated efforts [14].

Push efforts are often researcher-led and include efforts to disseminate user-friendly knowledge or seek policy-relevant research questions [14]. User pull efforts include activities to support policy-makers in searching and using evidence for decision-making [14, 17, 18]. Exchange efforts involve fostering joint interaction, collaboration and partnerships between research producers and users [14, 17]. Integrated efforts incorporate pull, push and exchange efforts and are often facilitated through knowledge brokers (individuals or organisations/networks) that bring together policy-makers, researchers and other stakeholders to conduct KT activities [14, 17]. These activities outlined in the framework largely contribute to KT by building capacity (i.e. skills development and continuing education) and the development of KT infrastructure [14]. For the purposes of this study, we adopted the Ellen et al. [19] definition of research knowledge infrastructure to define KT infrastructure as any instrument (i.e. programmes, interventions, tools, devices) used to facilitate the access, dissemination, exchange and/or use of evidence.

Integrated efforts using organisational knowledge brokers to facilitate KT are widely used by global policy actors to develop capacity and infrastructure at the country-level [6, 17, 20–22]. Organisational knowledge brokers involve the development of multisectoral bodies comprised of researchers, policy-makers and other stakeholders to collaboratively facilitate KT [17, 23]. One such initiative is the multi-year programme, Building Capacity to Use Research Evidence (BCURE) developed by the Evidence for Policy Design from the Harvard Kennedy School in collaboration with United Kingdom Aid [24]. The BCURE programme developed organisational knowledge brokering projects across 12 low- and middle-income countries from 2013 to 2017 and built KT capacity in over 560 stakeholders [20]. A similar initiative from the McMaster Health Forum in Canada, the Partners for Evidence-driven Rapid Learning in Social Systems (PERLSS), is currently working to establish organisational knowledge brokers and strengthen KT capacity in 13 partner countries, with the aim of supporting countries in achieving the Sustainable Development Goals [21, 25].

Recognising that many countries face low KT capacity, coupled with requests from Member States to develop innovative mechanisms for bridging the research-to-policy gap, the WHO launched its organisational knowledge brokering initiative in 2005 called the Evidence-informed Policy Network (EVIPNet) [6, 26, 27]. Operating at the global, regional and national levels, EVIPNet’s aim is to support national policy-makers, researchers and other stakeholders to systematically and transparently use high-quality evidence in policy-making [6]. EVIPNet establishes country-level organisational knowledge brokers, so-called KT Platforms (KTPs) under the EVIPNet terminology, as a means to build capacity and institutionalise KT infrastructure in network participant countries [17, 23]. EVIPNet’s KTPs develop products such as rapid response services, evidence briefs for policy and policy dialogues, and conduct capacity-building exercises with national stakeholders to retrieve, assess, synthesise, package and use evidence [6].

The European arm of EVIPNet (EVIPNet Europe), for example, launched under the umbrella of the WHO European Health Information Initiative, has supported 21 network participant countries in KT capacity-building and infrastructure development since its inception in 2012 [28]. Network members have developed a range of KT instruments in recent years that catalyse policy change at the national level and several countries have successfully implemented preliminary steps to institutionalise KTPs [28, 29]. By improving health policy-making processes, EVIPNet Europe supports the implementation of regional and global policy goals such as the Health 2020 European policy framework [30], the Action Plan to Strengthen the Use of Evidence, Information and Research for Policy-making in the WHO European Region [31] and the Sustainable Development Goals [25].

While there has been some evaluation of these efforts, mainly from programme leadership [28, 32, 33], challenges related to the low capacity of organisational knowledge brokers to evaluate their own activities have been noted [16]. Low capacity coupled with the complexity of policy-making processes, which are often influenced by a number of factors that make attributing policy and health outcomes to any one aspect difficult, can create challenges for organisational knowledge brokers to evaluate their work [34, 35]. Using high-quality, evidence-informed indicators can support organisational knowledge brokers and ensure greater attribution to their efforts [36]. While impact evaluation is important and work has been done in this area [37, 38], focusing on shorter and intermediate evaluation indicators is more likely to result in greater attribution to KT activities [39]. For this reason, our study focuses solely on output indicators (measure programme outputs including products, goods and services resulting from an intervention) and outcome indicators (measure the short- and medium-term effects of an intervention’s outputs) [40].

While there has previously been a lack of indicators to evaluate KT activities [41, 42], some work has been done in this area in recent years [38, 43]. For example, Tudisca et al. [38] have developed 11 indicators to assess evidence use in policy-making using a Delphi study; however, the study does not include indicators to assess KT activities. Maag et al. [43] have collected and assessed indicators for measuring the contributions of knowledge brokers but the study is not specific to health policy and focuses on individual knowledge brokers; consequently, it lacks indicators to assess the development of KT infrastructure at the country level, which is a main aim of the organisational knowledge brokers currently active in the global health policy arena. The aim of this study was to identify, synthesise and assess indicators that have been used to evaluate KT infrastructure and capacity-building activities, in order to support organisational knowledge brokers and their stakeholders in evaluation.

## Methods

We have used the scoping review methodology as outlined in the Arksey and O’Malley [44] framework. This included (1) identifying the research question; (2) identifying the relevant studies; (3) determining the study selection; (4) charting the data; and (5) collating, summarising and reporting the results [44]. The following research questions guided the review: What indicators have been used to measure KT infrastructure and capacity-building in evaluation literature? What percentage of indicators are based on previously developed methods or are validated?

A search strategy was developed, piloted and refined in consultation with a team of medical librarians at Karolinska Institutet (see Additional file 1 for full search strategy). Three databases were used, namely Ovid MEDLINE, Ovid Global Health and the WHO Library Database – a combination that searched both peer-reviewed and grey literature. Additional literature was collected via reference searching of eligible articles and manual addition by the authors.

### Inclusion and exclusion criteria

To capture the indicators relevant for organisational knowledge brokers, we included studies evaluating KT infrastructure or KT capacity-building. The inclusion criteria consisted of (1) studies published in English language, from January 1, 2005, through December 31, 2016 (the year 2005 was used as the lower cut-off year for the search since investments into increasing the linkages between research and policy substantially increased globally with World Health Assembly 58 resolution [5]); (2) studies that evaluated KT infrastructure or capacity-building efforts between research and policy-making, only. Due to the heterogeneity of the topic, the review was limited to the research–policy gap, though other forms of KT between researchers, communities, patients and clinicians could contribute useful indicators and/or perspectives; and (3) studies conducted on the macro scale, which was defined as an administrative geographical level of policy-making or research activity occurring at the national or supranational level.

All types of study designs were eligible for inclusion. Studies were excluded from analysis if they (1) did not evaluate or develop an evaluation framework for a KT infrastructure or capacity-building intervention or mechanism for increasing KT or evidence-informed policy-making (EIP); (2) discussed KT infrastructure or capacity-building in individual clinical decision-making or described capacity to implement evidence-based interventions or capacity-building efforts to front-line staff; (3) described or evaluated KT tools and products without focusing on infrastructure or capacity-building; (4) described capacity-building efforts with no intervention regarding KT or researcher–policy interaction; (5) described indicators to measure capacity, without a capacity-building effort implemented or described; (6) described a KT capacity-building effort between researchers and policy-makers at the sub-national level (however, some cases that fell into this distinction were included as they were deemed to be equally autonomous to the national level given the unique situation of the country); (7) focused on community-based participatory research, incorporating community needs in interventions or policy; (8) described the dissemination of evidence-based interventions or of quality improvement interventions; (9) focused on coalition and network best practices for achieving a goal besides KT; (10) were studies that fell under the category of implementation science (while similar and even complementary to KT, implementation science focuses more on barriers and facilitators of delivering an intervention, rather than the processes of evidence use [45]); or (10) had no published abstract.

All authors were involved in developing the search criteria and data extraction tool. Article screening, data extraction and charting were led by one reviewer (JS) and verified with a second reviewer (ZEK) when eligibility was unclear. The final references included for analysis and the extracted data were presented to and reviewed by all authors. While having two independent reviewers is ideal, the literature notes that the scoping review methodology can be adapted for feasibility, for example, by using one reviewer and a second reviewer to verify the data [46]. This modified approach was most feasible for our study given the available resources.

Screening of the collected data was guided by the Preferred Reporting Items for Systematic Reviews and Meta-analyses (PRISMA) flowchart [47], where first duplicate records were removed and then screened by title and abstract. For those abstracts for which eligibility was uncertain, full text articles were reviewed. Included articles were then reviewed in their full text. The following data items were extracted from the included literature and charted for analysis: title, author, year, country, study design, evaluation method, outputs and indicators, outcomes and indicators, mode of verification, duration and/or frequency of collection, and indicator methods. The domains of the Lavis et al. [14] research-to-action framework were used to categorise the output and outcome indicators. The conditions for each domain, that if met would likely be conducive for effective KT, were also used to guide our suggestions for applying the indicators (Table 1) [14].

**Table 1:** Indicator domain descriptions, adapted from the Lavis et al. [14] framework

Domain	Description	Conditions conducive to KT
General climate	Explores the extent to which funders and other stakeholders (i.e. universities, researchers and users of research) value and/or support efforts to link research to action	• Funder mandate to support KT activities • Universities and research institutions consider KT in their tenure/promotion process • Researchers value the promotion and use of evidence • Research users value evidence and its use
Production of research	Explores the extent to which research is produced in a way that is aligned with policy priorities; this domain suggests that researchers engage in priority-setting to ensure that users’ needs are identified and then develop scoping reviews, systematic reviews and single studies to address these needs	• Funders engage potential evidence users to identify policy needs and priorities • Funders and ethics review boards value systematic reviews
Push efforts	Includes efforts to create action based on messages arising from research; this domain includes developing actionable messages for end-users, disseminating research results and building research capacity to conduct these strategies	• Regularly identify actionable messages from systematic reviews • Develop user-friendly messages from evidence • Work with credible messengers for each user group • Use research-informed strategies to encourage and support action based on the messages • Evaluate their KT efforts
Pull efforts	Includes efforts by end-users to acquire, assess, adapt and apply research; this domain includes capacity-building efforts for policy-makers to use research	• Engage in the self-assessment of abilities to acquire, assess, adapt and apply research • Develop structures and processes to help them use and promote research
Exchange efforts	Includes efforts to develop partnerships between researchers and users, and the extent to which the partnerships jointly address relevant questions; this domain includes KT tools that facilitate exchange like policy dialogues	• Ongoing relationship building to develop an understanding of the cultural and other differences between the contexts of researchers and research users • Creations of meaningful partnerships where the roles and expertise of both researchers and research users are recognised
Integrated efforts^a^	This is not a domain in the Lavis et al. framework; we have adapted the original domain ‘efforts to facilitate user pull’ and expanded this to integrated efforts, which we define as using brokering to facilitating push, pull and exchange efforts; recognising that the push, user pull and exchange efforts are not mutually exclusive, integrated efforts aim to institutionalise KT infrastructure to facilitate a combination of activities encompassed in all three of these models of KT [17]	• Multidisciplinary leadership, comprised of researchers, funders and policy-makers, that are guided by a clear goal • Regular priority-setting processes • Facilitate and conduct push efforts using actionable messages • Engage in a variety of efforts to facilitate KT (e.g. one-stop shopping resource of relevant and quality systematic reviews, rapid-response unit to provide evidence summaries) • Facilitate exchanges between research producers and research users (e.g. policy dialogues) [17] • Develop and sustain KT infrastructure by institutionalising organisational knowledge brokers [17]
Evaluation	Explores the extent to which stakeholders participate in evaluating their KT activities; this domain also concerns assessing sustainability of KT initiatives	• Funding is allotted for evaluation of KT efforts • Funders, researchers, intermediary groups and user groups participate in rigorous evaluations of efforts to link research to action
Capacity-building^a^	Includes efforts to improve stakeholder capacity in any of the KT models (push, pull and exchange) and explores the extent to which these activities were successful in skills development; capacity-building is an element in many of the Lavis et al. domains but is not a domain of the original framework; it was adapted for use in this study	• Researchers partake in continuing education programmes to develop KT skills (e.g. systematic reviews, priority-setting)^b^ • Researchers partake in skills-development programmes to build capacity for developing and executing push efforts^c^ • Research users partake in skill-development programmes to build capacity for acquiring, assessing, adapting and applying research^d^ • Capacity-building programmes to support researchers and research users to engage in mutually beneficial partnerships^e^ • Stakeholders (e.g. funders, researchers or intermediary groups) partake in skills-development programmes to build capacity to develop and execute efforts to facilitate KT^f^

*KT* knowledge translation

^a^Denotes domains that are not included in the Lavis et al. framework

^b^Originally presented under the production of research domain in the Lavis et al. framework

^c^Originally presented under the push efforts domain in the Lavis et al. framework

^d^Originally presented under the user pull efforts domain in the Lavis et al. framework

^e^Originally presented under the exchange efforts domain in the Lavis et al. framework

^f^Originally presented under the efforts to facilitate user pull efforts domain in the Lavis et al. framework; it has been adapted to include capacity-building around efforts to facilitate KT in general, not only user pull efforts

Indicator methods were assessed by the extent to which they were informed by evidence, where expert opinion was considered least rigorous and validation procedures as most rigorous. Indicators that were informed by previously published frameworks, tools or literature, or developed using qualitative methods, were deemed to have been informed and included. When indicator sources were not mentioned or authors failed to provide a comprehensive description of indicator methods, the indicators were not deemed to be informed and were excluded.

No ethical approval was necessary to conduct this study, as it collected publicly published reports.

## Results

### Study characteristics

**Fig. 1:**
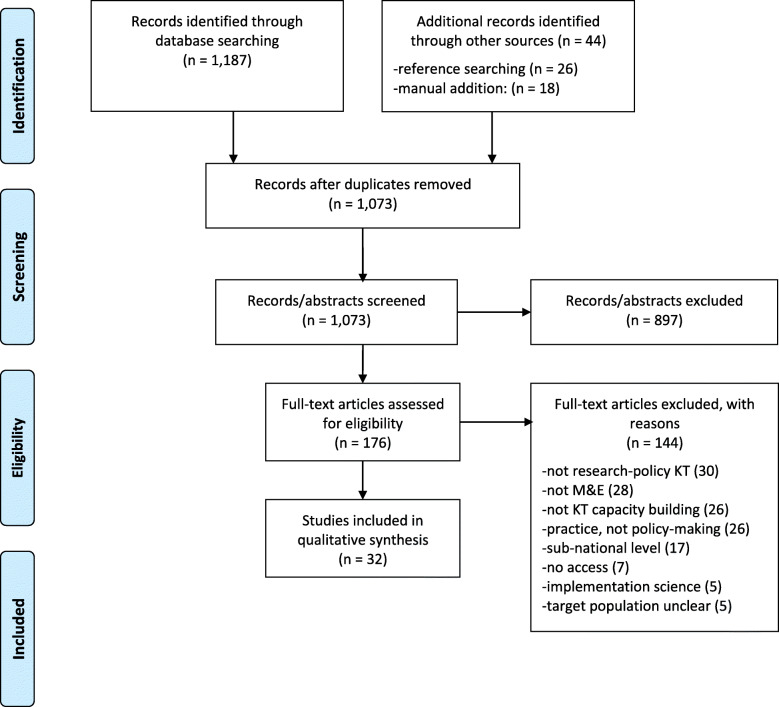
Study selection flowchart according to Preferred Reporting Items for Systematic Reviews (PRISMA)

Of 1231 articles obtained from the database search, reference searching and manual addition, 32 met the inclusion criteria and were included in this study. The full study selection process with reasons for exclusion is outlined in Fig. 1.

Of the 32 eligible studies, 3 articles were study protocols for a randomised control trial (RCT), 3 studies were general evaluation frameworks and 1 study developed indicators for use in monitoring and evaluation (M&E). The remaining 25 studies were programme/intervention evaluations. Participants and target audiences were mainly policy-makers (*n* = 21/32) and researchers (*n* = 17/32). Other targeted populations included the public sector, academic and research institutions, healthcare personnel and institutions, non-governmental organisations and advocacy groups, development agencies, media, donors and funders, and civil society. More details of study characteristics can be seen in Additional file 2.

Of the 32 studies, integrated efforts and push efforts were the most represented with 9 studies each. Specific integrated efforts included KT brokering (*n* = 4), secretariat technical assistance (*n* = 1), KTPs/organisational knowledge brokers (*n* = 3) and research networks (*n* = 1). Push efforts included research funding (*n* = 4), research platforms (*n* = 2), researcher capacity-building academic programmes (*n* = 1), complex interventions (*n* = 1) and research partnerships (*n* = 1). Seven studies (*n* = 7/32) represented linkage and exchange efforts, which included research partnerships and platforms (*n* = 3), KT and knowledge exchange networks (*n* = 2), buddying (*n* = 1), and complex systems interventions (*n* = 1). User pull activities included workshops (*n* = 2), a workshop with mentoring (*n* = 1), a conference (*n* = 1), an organisational complex intervention (*n* = 1), online resources (*n* = 1) and technical assistance (*n* = 1). Capacity-building efforts were found to be mainly focused on producer push and user pull, whereas infrastructure was mainly focused on exchange and integrated efforts. The main KT infrastructural tools included platforms, partnerships, networks, and KTPs or other organisational knowledge brokers.

### Indicators organised by domain

All 32 studies reported outputs, with fewer reporting outcomes (*n* = 26/32). Output and outcome indicators were organised using the domains included in the Lavis et al. [14] framework for assessing country-level efforts to link research to action. One domain from the original framework, ‘efforts to facilitate user pull’, was revised to ‘integrated efforts’ for the purposes of this study. This revised domain includes facilitation not only of pull efforts but also of push and exchange efforts, since organisational knowledge brokers facilitate KT across all three activities [17]. ‘Capacity-building’ was also added as an additional domain. Capacity-building is included in the Lavis et al. [14] framework but is not a separate domain. We chose to highlight these indicators in a dedicated section since they apply to many of the other domains. Table 1 includes a full description of the eight domains used to categorise the collected indicators and the conditions outlined in the framework that, if met, would likely be conducive for KT.

In total, 213 indicators were identified, including 181 output indicators and 32 outcome indicators. Of the 213 indicators identified, 174 (81.7%) were based on methods beyond expert opinion (i.e. literature review, frameworks, published tools) or had been validated. Of these, 155 were output indicators and 19 were outcome indicators. Indicators that were based on literature review, pre-existing frameworks or were validated are presented in Table 2. Most studies developed indicators specifically for the evaluation highlighted in the study and used non-systematic literature review, theoretical frameworks, published tools and/or expert opinion. One study developed indicators using literature review and focus groups with stakeholders, one study developed and validated indicators, and three studies used previously validated indicators.

**Table 2:** The 174 method-based indicators to evaluate organisational knowledge brokers

1. General climate	
Output indicators	
1.1 Number of activities identified 1.2 Availability of synthesised and packaged evidence 1.3 The organisation has the skills, structures, processes and a culture to promote and use research findings in decision-making^a^ 1.4 Feedback on context/culture 1.5 Facilitators, barriers, lessons learnt	
Outcome indicators	
1.6 Increased demand or value of KT products or knowledge from policy-makers 1.7 Number of times evidence is mentioned in policy/parliamentary discussions 1.8 Increased awareness of importance of EIP initiatives 1.9 Changes in government allocated funding	
2. Production of research	
Output indicators	
2.1. Number of peer-reviewed journal articles 2.2. Citations per article 2.3. Citation of research results by other researchers 2.4. Journal impact factor 2.5. Number of projects per research approach 2.6. Funds invested per project 2.7. Project duration 2.8. Number of projects liaising with users 2.9. Number of projects that led to subsequent research 2.10. Researcher feedback on project alignment with priorities 2.11. Mean score of scientific accuracy	2.12. Mean score of readability 2.13. Mean score of usability 2.14. Mean score of ease of access 2.15. Applicability of research for decision making 2.16. Developed priority report (i.e. research agenda, list of priorities, country assessment) 2.17. Revision with stakeholders 2.18. Feedback on support and/or awareness 2.19. Feedback on priority development 2.20. Feedback on priorities
Outcome indicators	
2.21. Changes in policies or programmes consistent with evidence produced 2.22. Policy-makers, stakeholders and researchers report that relevant and understandable health research evidence is more readily available and cite this research evidence in media	
3. KT activities: push efforts	
Output indicators	
3.1. Number of downloads 3.2. Number of page visits (total and unique) 3.3. Number of countries visiting the website 3.4. Number of page views per visit 3.5. Number of requests for materials 3.6. Extent of media exposure 3.7. Referrals made to distributed materials 3.8. Number of materials distributed 3.9. Transmitted to relevant stakeholder (discussed at policy dialogues, dissemination workshops) 3.10. Disseminated materials are read and understood 3.11. Efforts have been made to adopt the disseminated knowledge 3.12. Platform survey responses 3.13. Usage analytics of promotional products 3.14. Research is presented to decision-makers in a useful way^a^ 3.15. Multiple formats of written and/or other forms of presentation (e.g. newsletter, website summary, interim report, oral presentation) 3.16. Presentation formats include layman’s terms and recommendations 3.17. Where appropriate, presentation formats are concise (e.g. less than two pages)	3.18. Users contacted researchers to discuss results 3.19. Relevant documents disseminated in hardcopy 3.20. Website or online evidence database is established 3.21. Number of dissemination workshops 3.22. Percentage of grantees presenting at conferences 3.23. Percentage of grantees submitting work for publication 3.24. Percentage of grantees with published research at time of review 3.25. Feedback from grantees on competence and opportunities for dissemination 3.26. Number/amount of grant (applications) 3.27. Number of researcher internships 3.28. Number of trainees publishing research 3.29. Feedback on improved quality of research results 3.30. Percentage of research applications headed by a national 3.31. Increased interest by young nationals in research 3.32. Establishment of a PhD programme 3.33. Number of projects supported
Outcome indicators	
3.34. Number of project findings used/expected to be used in policy 3.35. Number of projects leading to/expecting to change behaviour 3.36. Increase in inquiries and applications 3.37. Phasing out of external funding	
4. KT activities: pull efforts	
Output indicators	
4.1. Seeking, Engaging with, and Evaluating Research (SEER)^a^ 4.2. Organisational Research Access, Culture and Leadership (ORACLe)^a^	
Outcome indicators	
4.3. Staff Assessment of enGagement with Evidence (SAGE)^a^	
5. KT activities: exchange efforts	
Output indicators	
5.1. Grants for collaboration 5.2. Research projects are produced with policy-makers 5.3. Disciplinary backgrounds of contributing authors 5.4. Invitations to publish special issues 5.5. Partners views on using research results 5.6. Negotiation occurs during the research process 5.7. Negotiated items are clearly understood by all 5.8. Deciding on objectives together 5.9. Built mutual trust 5.10. Communication tools established 5.11. Sharing of information and responsibility 5.12. Transparency 5.13. Share profits equally 5.14. Build on achievements 5.15. Communication is clear 5.16. Communication is relevant 5.17. Communication is timely 5.18. Communication is respectful 5.19. Density and centrality 5.20. Connectedness of networks 5.21. Partners mention each other 5.22. Partners are flexible about meeting partner’s changing needs and revising research plans and timelines 5.23. Partners understand the limits of each other’s flexibility 5.24. Partners understand research findings, their limits and their implications for Ministry work 5.25. Conflict is dealt with openly, informally and promptly	5.26. Trust has increased between partners 5.27. Comfort has increased between partners 5.28. Openness has increased between partners 5.29. Partners begin speaking a common language regarding research 5.30. Partners facilitate removal of barriers for each other’s work 5.31. Linkage with partner enhances partner linkage with community/other stakeholders 5.32. There is joint commitment to the research project 5.33. There is an increase in joint activity around the project 5.34. Clear leadership of partnerships 5.35. Team mentality 5.36. Early engagement of members 5.37. Number of members 5.38. Number/percentage of members present at activities 5.39. Level of engagement 5.40. Number/percentage of partners active 5.41. Member affiliation and profession 5.42. Joint meetings occur at most stages of research 5.43. Joint meetings occur to discuss research dissemination and utilisation plans 5.44 Feedback on linkage and exchange mechanisms 5.45. Number of partners involved in KT activities 5.46. Stakeholders involved
Outcome indicators	
5.47. Partners are perceived as experts in the research/policy area and are referred to as such to others 5.48. Value of network 5.49. Feedback on awareness and perceptions of network 5.50. Partnerships are built and sustained	
6. KT activities: integrated efforts	
Output indicators	
6.1. Number of KTPs viewing their work as a long-term initiative 6.2. Number of KTPs engaging in priority-setting with stakeholders 6.3. Number of KTPs building capacity for priority-setting 6.4. Number of KTPs producing/in process of KT products (by type, e.g. evidence briefs, clearinghouses, rapid response services, deliberative dialogues, systematic reviews) 6.5. Number of KTPs that built capacity for KT (evidence briefs; deliberative dialogues; accessing, assessing and using research evidence) 6.6. Number of KTPs training research users in KT (systematic reviews, evidence briefs, deliberative dialogues) 6.7. Number of organisations using the products 6.8. Functional website or clearinghouse providing KT resources 6.9. Amount of resources utilised in knowledge brokering activities (e.g. cost, time, materials) 6.10. Number of KT materials 6.11. Products (e.g. website, policy dialogues, evidence briefs) aligned with and address priorities	6.12. Topic of KT materials 6.13. Number of KT materials translated/available in different languages 6.14. Policy dialogues about high-priority policy issues take place regularly 6.15. Scoring of quality dimensions (mean, standard deviation) 6.16. KT activities regarded as beneficial for bringing together stakeholders and facilitating the development of partnerships
Outcome indicators	
6.17. Uptake and/or influence of evidence (reports, policy briefs, recommendations, other) in decision-making 6.18. Financial and organisational support to the KTP	
7. Evaluation	
Output indicators	
7.1. Number of KTPs evaluating KT product(s) quality 7.2. Perception of sustainability (no outcome indicators identified)	
8. Capacity-building	
Output indicators	
8.1. Number of activities 8.2. Type of activity 8.3. Number of people invited 8.4. Number of people attended 8.5. Number of people trained 8.6. Reasons for participation non completion 8.7. Participant occupation 8.8. Participant affiliation 8.9. Participant education level 8.10. Participant gender 8.11. Participant age 8.12. Participant’s number of years in current position 8.13. Participant’s level of policy influence 8.14. Country of participants 8.15. Participant’s experience with evidence-informed policy-making 8.16. Training workshops for policy-makers and researchers are designed and implemented regularly 8.17. Mean programme ratings and feedback 8.18. Intent to return 8.19. Survey response rate 8.20. Percentage increase in pre/post scores of skill abilities (e.g. access evidence, synthesise evidence, policy dialogues, evidence briefs, collaboration etc.) and value of knowledge use	8.21. Comments in the media reflect capacity changes 8.22. Ability to acquire research 8.23. Increased research capacity 8.24. Change in research/policy-maker relationship 8.25. Comments in the media reflect relationship changes 8.26. Perceived EIP skills/changes in skills (acquire, assess, adapt, apply)^a^ 8.27. Number of participants reporting benefits 8.28. Awareness of key government documents 8.29. Perceived change in skills and confidence to interact with experts 8.30. Perceived impact on current position and/or future career advancement^a^ 8.31. Contribution to decision-making by partners and policy-makers
Outcome indicators	
8.32. Feedback on behavioural changes 8.33. Number/percentage of trainees reporting intent to use skills gained	

*EIP* evidence-informed policy-making, *KT* knowledge translation, *KTP* knowledge translation platform

^a^Denotes indicators that have validated measurement tools

Four of the 32 studies used the validated indicators (as noted by an ^a^ in Table 2). Three of the four studies were part of the same project, which used indicators from the validated Is Research Working for You? tool [48–50]. Other validated tools included the Staff Assessment of engagement with Evidence (SAGE), Seeking, Engaging with, and Evaluating Research (SEER), and Organisational Research Access, Culture and Leadership (ORACLe) [41]. These indicators were mainly used to evaluate capacity, not KT infrastructure. They are derived from both qualitative and quantitative methods, using interviews or questionnaires as evaluation methods. As a capacity measure, these indicators were used in pre/post interventions as both a baseline and outcome measures [41, 48–50].

**Table:** 

Measure descriptions of the four validated indicators and tools:	
• ORACLe Measures: capacity (policies that encourage or mandate the examination of research in policy and programme development; tools, systems, training and programmes to assist with accessing, appraising and generating research).	
• SEER Measures: capacity (value placed on research, perceived value organisation places on research; confidence in skills and knowledge to access, appraise and generate research; perceived availability of organisational tools, systems, training and programmes to assist with accessing, appraising and generating research); research engagement actions (self-reported extent of accessing, appraising and generating research, and interaction with researchers); research use (self-reported use of research).	
• SAGE Measures: research engagement actions (accessing research, appraising research (for quality and relevance), generating new research or analysis, and interacting with researchers) and research use (four types of research use are considered: instrumental, tactical, conceptual and imposed) in each policy document and the context in which the policy document was produced, including barriers and facilitators.	
• Is Research Working for You? Tool This tool was developed in 2009 by the Canadian Foundation for Healthcare Improvement (formerly the Canadian Health Services Research Foundation) and includes 88 items to measure culture for EIP and use of evidence at the individual and/or organisational level.	

## Discussion

We identified 213 unique output and outcome indicators related to KT infrastructure and capacity-building, of which 174 were based on methods beyond expert opinion. Few of the indicators had been validated or assessed for rigor and many studies did not report methods for selecting indicators at all. The literature notes the common trade-off that exists between collecting data with quality indicators versus collecting data that is already being used for monitoring [40], which may be a driving factor that many indicators were not explicitly described or based on methods. The indicators measured using the four validated tools (SEER, SAGE, ORACLe and Is Research Working for You? [41, 48–50]) should be highlighted as the most rigorous indicators collected in this review.

To the best of our knowledge, this study is the first to identify indicators to evaluate KT infrastructure and capacity-building activities specific to organisational knowledge brokers in a health policy-making context. Maag et al. [43] and Tudisca et al. [38] have both published indicator lists, the former to assess the contributions of individual knowledge brokers and the latter to assess the use of evidence in policy-making. Our study is distinguished from these other indicator studies as we collected indicators to assess organisational knowledge brokers in their work to build capacity and KT infrastructure. It is also distinguished from the Gagliardi et al. [51] scoping review that synthesised 13 evaluation studies of integrated KT activities across varied healthcare settings since our study extracted and assessed evaluation indicators. The indicators synthesised in this study are both generalisable and transferable to other KT infrastructural and capacity-building efforts at the national and regional levels, given the broad eligibility criteria. However, since the indicators presented in this synthesis are broad indicators that should be adapted to the specific needs and activities of KT stakeholders [52], the transferability and generalisability of the indicators may not affect their practical use.

### Applying the indicators in practice

The summary list of indicators can guide the evaluation of organisational knowledge brokers, such as the KTPs implemented under EVIPNet as well as similar capacity-building initiatives that operate via organisational knowledge brokers [20–22]. For example, this list has been presented to the WHO Secretariat of EVIPNet Europe and has already been used in EVIPNet Europe’s mid-term evaluation and M&E framework. We have also provided data on mode of verification, method and frequency of collection for each indicator in Additional file 3 to guide stakeholders in applying them in practice.

In selecting indicators to assess country-level KT indicators, we suggest stakeholders use the Lavis et al. [14] framework as a guide. In particular, the conditions outlined under each domain that, if met, would likely be conducive with linking research to action; these conditions are outlined in Table 1 above. Additionally, there are several factors that should be carefully considered when selecting indicators for M&E. Current evaluation literature recommends using a mix of both qualitative and quantitative indicators [52]. Qualities such as validity, acceptability, feasibility, sensitivity and predictive validity should also be considered [40]. The related costs and resources required for the collection of an indicator are also determining factors in selecting M&E indicators and, often, such factors may result in the use of indicators based on existing data or data collection instruments over the ‘best fit’ indicator [40]. Selecting fewer but essential and higher quality indicators can mitigate such trade-off [40]. Based on these considerations, we outline a few examples of using the presented set of indicators to evaluate organisational knowledge brokers. The two examples highlight that, for some activities, there may be one composite indicator available (when multiple indicators are compiled into a single index [53]) and, for other activities, stakeholders may need to select a variety of indicators based on the type of activity they are evaluating.

To assess the general KT climate, for example, a programme may be interested in evaluating current outputs or outcomes of KT activities. Since there is a validated output indicator to assess the general climate, we suggest that stakeholders use the indicator ‘the organisation has the skills, structures, processes and a culture to promote and use research findings in decision-making’, which is collected using the Is Research Working for You? tool [48–50]. This indicator will assess most of the considerations detailed in the Lavis et al. [14] framework that include structural supports and individual value of research use. The indicator can be used to assess both organisations and individuals, and can be used both as a pre-test assessment to understand the current state of the KT climate or as a post-test assessment to understand if KT activities have contributed to an improved climate [48–50]. Similarly, this study collected three composite indicators – SEER, ORACLe and SAGE – that can be used to evaluate both the outputs and outcomes of pull efforts. These indicators can also be used as pre/post-test assessments [41].

On the other hand, to assess push efforts, stakeholders may need to use a variety of indicators since there is no one indicator that captures all conditions outlined by Lavis et al. [14]. The condition of developing user-friendly messages from the evidence can be evaluated using a validated indicator, ‘research is presented to decision-makers in a useful way’, which is also measured using the Is Research Working for You? tool [48–50]. However, stakeholders may also be interested in assessing their outputs of strategies employed to encourage the use of evidence. To do so, we recommend using a few output indicators that apply to the strategy taken. For example, if an organisational knowledge broker disseminated research findings and actionable messages using a website, it may be useful to collect data on the number of downloads, number of page visits and number of countries visiting the website since these three indicators will provide a snapshot of reach and level of engagement. Outcomes of these strategies are also likely of importance to stakeholders. The four outcome indicators collected to assess push efforts all capture distinct items: use of research, behaviour change, increased interest in KT from researchers and changes in funding. The first two indicators, ‘number of project findings used/expected to be used in policy’ and ‘number of projects leading to/expecting to change behaviour’ would demonstrate the effect of efforts on evidence use, while the latter two indicators, ‘increase in inquiries and applications’ and ‘phasing out of external funding’ would rather demonstrate the effect of efforts on sustainability. Selecting which indicator would be best and how many to use would ultimately depend on the type of work being assessed, the goals of the evaluation, data availability and resources.

### Limitations and further research

The findings are limited in scope since only articles published in English were included. Ensuring a comprehensive list of search terms was important to minimise bias in study representation towards a particular country, region, organisation or funder given that the KT terminology used varies widely by such factors [54]. Careful attention was paid to develop a comprehensive search strategy, which was informed by a literature search and piloted several times in consultation with medical librarians. The use of only two databases, both of which are health focused, may have excluded relevant studies from other disciplines. However, the peer-reviewed database search was supplemented using grey-literature databases, reference searching and manual addition.

Despite developing inclusion and exclusion criteria, decisions on eligibility often needed to be interpreted due to the complexity and heterogeneity of the topic. While only one reviewer screened and analysed the data, a second reviewer was consulted where eligibility was unclear. We acknowledge this as a limitation of our work, since having two reviewers is ideal under the Arksey and O’Malley framework [44]. However, our modified approach using one reviewer and a second reviewer to verify results has been used by other stakeholders in the field and was carefully developed to be robust while still being feasible for our study given the available resources [46]. The challenges faced due to the complexity of the KT field further emphasise the previous calls for action on developing a more uniform KT vocabulary and adding to existing efforts [55]. Due to a widespread lack of detail regarding evaluation and indicator methods, some rigorous or method-based indicators may have been excluded. Many studies did not explicitly state which indicators were used as output or outcome indicators and were then categorised by judgement of the reviewer.

Further analyses using the Delphi method and stakeholder interviews can contribute to our findings regarding the comprehensiveness, acceptability and feasibility of the presented indicators [40]. A complementing review to identify institutionalisation indicators to assess the extent to which organisational knowledge brokers have been systematically integrated into national health policy-making would be useful. By organising the collected indicators with the Lavis et al. [14] framework domains we have also identified opportunities for further work with indicator development and validation. For example, there was a minimal number of indicators identified to assess programme’s evaluation efforts. This may be due to a potential lack of evaluation occurring in the field. Additionally, this review found no validated indicators that have been used to evaluate exchange efforts. The literature on the institutionalisation of organisational knowledge brokers is also scarce and further work, including developing institutionalisation frameworks, would aid the development of institutionalisation indicators.

M&E provides valuable insight for quality improvement and can help strengthen organisational knowledge brokers in their work to make the policy-making process more systematic and transparent. This is particularly important since policy-making is a complex process, often influenced by political will and obligation, civil society needs and opinion, cultural norms, resource considerations, lobbying and advocacy [34, 35]. Strong KT infrastructure provided by organisational knowledge brokers can support more equitable and efficient policies [56]. Demonstrating the effectiveness of organisational knowledge brokers through M&E is particularly important for sustainability, as it can lead to greater funder interest and buy-in. Strong support for organisational knowledge brokers will help countries work towards the EIP goals they have agreed to [4, 5, 7] and assist them in meeting current policy targets [30].

## Conclusion

The gap between research and policy is a result of several competing factors, one of which is a lack of capacity for EIP [27]. More specifically, many countries do not have the structural capacity to systematically and transparently use high-quality evidence. Efforts to support countries in developing such systems using organisational knowledge brokers have been implemented in cooperation with several global and local stakeholders. WHO’s EVIPNet, as an example, works collaboratively with countries to develop organisational knowledge brokering platforms comprised of researchers, policy-makers and other stakeholders [6].

M&E is vital for ensuring the success and sustainability of organisational knowledge brokers. However, resources for evaluating organisational knowledge brokers in a health policy context are limited. As organisational knowledge brokers are implemented and become more established, it is important to build stakeholder capacity to evaluate their work. This review presents a total of 174 method-based indicators to evaluate KT infrastructure and capacity-building. Four validated instruments, namely SEER, SAGE, ORACLe and Is Research Working for You? [41, 48–50], were also identified. While this study provides a critical starting point for future development of KT indicators, the presented indicators in their current form can be used or adapted globally by organisational knowledge brokers and other stakeholders in their M&E work.

## Supplementary information

**Additional file 1.** Search strategy used to search Medline and Global Health databases. This file presents the full search strategy used to search the Medline and Global Health databases.

**Additional file 2.** Study characteristics of the 32 eligible studies. This file details the complete list of included articles and includes data on the KT model of the KT infrastructure or capacity-building intervention, the country, the capacity-building level, the evaluation method used and the target audience.

**Additional file 3.** Indicator data extracted from eligible studies to inform their use in evaluation. This file has a complete list of output and outcome indicators based on methods beyond expert opinion that were extracted from the eligible studies. The table includes the indicators organised by domain, the modes of verification for the indicators, the method the indicators were based on or collected with, the frequency of collection, and the study the indicator was extracted from.

## Data Availability

Not applicable. Additional study data are provided in supplementary materials (see Additional files 1, 2 and 3).

## Abbreviations

EIP: Evidence-informed policy-making
EVIPNet: Evidence-informed Policy Network
KT: Knowledge translation
KTP: Knowledge translation platform
M&E: Monitoring and evaluation
ORACLe: Organisational Research Access, Culture and Leadership
PRISMA: Preferred Reporting Items for Systematic Reviews and Meta-analyses
SAGE: Staff Assessment of enGagement with Evidence
SEER: Seeking, Engaging with and Evaluating Research
