# Supplementary material for: Indicators to evaluate organisational knowledge brokers: a scoping review
Source: Health Res Policy Syst. 2020 Aug 24;18:93. doi: 10.1186/s12961-020-00607-8 (PMC7444249; doi:10.1186/s12961-020-00607-8)
Supplement: Supplementary file 1 — Additional file 1. Search strategy used to search Medline and Global Health databases. This file presents the full search strategy used to search the Medline and Global Health databases. [file 12961_2020_607_MOESM1_ESM.pdf]

Additional File 1 Search strategy used to seach Medline and Global Health databases

1. exp "Diffusion of Innovation"/

2. exp Information Dissemination/

3. ((knowledge or evidence-based or research*) adj2 (action or collaborat* or implement* or adopt* or applicat* or broker* or coproduction or diffusion or dissemination or exchang* or mobilization or mobilisation or network* or platform* or shar* or use* or using or synthes* or transfer* or translat* or uptake or utilization or utilisation)).ti,ab.

4. (evidence informed or knowledge intermediar* or "linkage and exchange").ti,ab.

5. 1 or 2 or 3 or 4

6. exp Decision Making/

7. exp Decision Making, Organizational/

8. exp Policy Making/

9. exp Health Planning Organizations/

10. (Decision mak* or Health systems strengthening or Policy* or Policies).ti,ab.

11. 6 or 7 or 8 or 9 or 10

12. exp Evaluation Studies/

13. exp Program Evaluation/

14. exp Capacity Building/

15. ((change* or measur* or monitor* or improv* or increas* or evaluat* or build* or achiev*) adj3 (capacity or evidence-informed or knowledge translation or knowledge use* or evidence use* or research use*)).ti,ab.

16. 12 or 13 or 14 or 15

17. 5 and 11 and 16
