# Supplementary material for: Indicators to evaluate organisational knowledge brokers: a scoping review
Source: Health Res Policy Syst. 2020 Aug 24;18:93. doi: 10.1186/s12961-020-00607-8 (PMC7444249; doi:10.1186/s12961-020-00607-8)
Supplement: Supplementary file 2 — Additional file 2. Study characteristics of the 32 eligible studies. This file details the complete list of included articles and includes data on the KT model of the KT infrastructure or capacity-building intervention, the country, the capacity-building level, the evaluation method used and the target audience. [file 12961_2020_607_MOESM2_ESM.pdf]

Additional File 2 Study characteristics of the 32 eligible studies

KT Model	Study	Country	KT capacity building activity/level	Evaluation method	Target audience
Push efforts	Aikins et al 2012: Establishing and sustaining research partnerships in Africa: a case study of the UK-Africa Academic Partnership on Chronic Disease	United Kingdom & Africa	Research partnership<br>-<br>Regional	[none reported]	researchers, policymakers
Push efforts	Angulo-Tuesta et al 2016: Impact of health research on advances in knowledge, research capacity-building and evidence-informed policies: a case study on maternal mortality and morbidity in Brazil	Brazil	Research funding<br>-<br>Systems	Case Study/Document review/citation analysis	Researchers
Push efforts	Bartlett et al 2008: 'I went in feeling like a student and came out feeling like a researcher': An evaluation of the first Australian Masterclass for Emerging Researchers in Ageing	Australia	Academic program<br>-<br>Individual	Pre/Post Intervention Questionnaire	researchers
Push efforts	Cole et al 2016: Implementing a national health research for development platform in a low-income country – a review of Malawi's Health Research Capacity Strengthening Initiative	Malawi	Research platform<br><br>-<br>Systems	Document review/Interviews	Researchers & research institutions
Push efforts	UNDP/UNFPA/WHO/WORLD BANK<br>Special Programme of Research, Development and<br>Research Training in Human Reproduction (HRP)	WHO Headquarters & participating countries (Ethiopia, Mozambique, Nigeria, Uganda, Zambia, South Africa, Kenya, Maldives, Fiji, Viet Nam,	Research platform<br>-<br>Regional	Case Study (lesson learning, interviews, document review, survey)	health-care providers, policy-makers, scientists, clinicians and consumer and community representatives

KT Model	Study	Country	KT capacity building activity/level	Evaluation method	Target audience
	2008: Knowledge synthesis and transfer: a case-study	the United Republic of Tanzania, China)			
Push efforts	Kwan et al 2007: A systematic evaluation of payback of publicly funded health and health services research in Hong Kong	Hong Kong, Special Administrative Region	Research fund<br>-<br>Systems	Research Payback Evaluation	professionals engaged in health research
Push efforts	Mahmood et al 2011: Strategies for capacity building for health research in Bangladesh: Role of core funding and a common monitoring and evaluation framework	Bangladesh	Funding and complex intervention<br><br>-<br>Organizational	Case Study	researchers & policymakers, donors, & management, academic institutions, development agencies
Push efforts	Ottoson et al 2009: Policy-Contribution Assessment and Field-Building Analysis of the Robert Wood Johnson Foundation's Active Living Research Program	The United States of America	Research funding<br>-<br>Systems	Questionnaire, Interviews, Document review, Bibliometric Analysis,	[none reported]
Push efforts	Sallis et al 2009: The Active Living Research Program: Six Years of Grantmaking	The United States of America	Research funding<br>-<br>Systems	Questionnaire, Interviews, Document review, Bibliometric Analysis,	[none reported]
User-pull efforts	Araujo de Carvalho et al 2015: Informing evidence-based policies for ageing and health in Ghana	Ghana	Technical assistance<br>-<br>Organizational	Case study/Document review	experts, policymakers, representatives from key ministries, the Ghana Health Service, teaching hospitals, professional bodies, NGOs and WHO.
User-pull efforts	The CIPHER Investigators 2014: Supporting Policy In health with Research: an Intervention Trial (SPIRIT)—protocol for a stepped wedge trial	Australia	Complex intervention<br>-<br>Organizational	Interviews, questionnaire, document review	Health policy agencies

KT Model	Study	Country	KT capacity building activity/level	Evaluation method	Target audience
User-pull efforts	Neves et al 2014: Evaluation of the international forum on evidence informed health policymaking: Addis Ababa, Ethiopia – 27 to 31 August 2012	Ethiopia	Conference<br>-<br>Individual	Post Intervention Questionnaire (qualitative & quantitative)	Researchers, policymakers, journalists
User-pull efforts	Peirson et al 2013: The Registry of Knowledge Translation Methods and Tools: a resource to support evidence-informed public health	Canada	Online resources<br>Individuals	Questionnaire, Interviews, Google Analytics	Policy & decision makers
User-pull efforts	Uneke et al 2011: Enhancing health policymakers' capacity to use information and communication technology in Nigeria	Nigeria	Workshop<br>-<br>Individual	Pre/Post Intervention Questionnaire	policymakers and other health decision makers, health researchers, NGOs
User-pull efforts	Uneke et al 2015: Improving Nigerian health policymakers' capacity to access and utilize policy relevant evidence: outcome of information and communication technology training workshop	Nigeria	Workshop<br>-<br>Individual	Pre/Post Intervention Questionnaire	policymakers
User-pull efforts	Uneke et al 2015: Enhancing the Capacity of Policy-Makers to Develop Evidence-Informed Policy Brief on Infectious Diseases of Poverty in Nigeria	Nigeria	Workshop & Mentoring<br>-<br>Individual	Pre/Post Intervention Questionnaire	policymakers
Linkage and exchange	Alberta Health Services 2014: Knowledge Tran1slation Evaluation Planning Guide	Canada	Research platform<br>-<br>Systems	[none reported]	Research partnerships
Linkage and exchange	ESSENCE on Health Research 2016: Planning, Monitoring and Evaluation	[General framework]	Research platform<br>-	Document review, site visits, questionnaires, interviews	funders of research capacity efforts

KT Model	Study	Country	KT capacity building activity/level	Evaluation method	Target audience
	Framework for Research Capacity Strengthening		Individual, Organizational, Systems		
Linkage and exchange	Galluzzo et al 2012: Alcohol and older people. The European project VINTAGE: Good Health into Older Age. Design, methods and major results	Europe	Network<br>-<br>Regional	Document review, questionnaire	healthcare professionals, policymakers, policy making organizations, researchers, academic institutions
Linkage and exchange	Hawkes et al 2016: Strengthening capacity to apply health research evidence in policy making: experience from four countries	Bangladesh, Gambia, Nigeria (India excluded; community level)	Complex intervention<br>-<br>Organizational	Document review, interviews, pre/post questionnaire	policymakers & policy influencers, researchers
Linkage and exchange	Kothari et al 2011: Indicators at the interface: managing policymaker-researcher collaboration	Canada	Research partnerships<br>-<br>Systems	not evaluation<br>indicator development through literature review, interviews, and focus groups	research partnerships
Linkage and exchange	Langlois et al 2016: Enhancing evidence informed policymaking in complex health systems: lessons from multi-site collaborative approaches	South Africa & Cameroon	Buddying<br>-<br>Individual	Realist Evaluation (Document review, interviews, focus group)	sub-national policymakers & researchers
Linkage and exchange	Murnaghan et al 2013: Knowledge exchange systems for youth health and chronic disease prevention: a tri-provincial case study	Canada	Network<br>-<br>Systems	Case Study, Lessons Learned (documents, surveys, interviews, focus groups)	stakeholders involved in practice, policy, or research
Integrated efforts	Conklin et al 2008: A Model for Evaluating Knowledge Exchange in a Network Context	Canada	KT Network & Brokering<br>-<br>Systems	Case study: Literature review, interviews, questionnaire	caregivers, policy-makers, researchers

KT Model	Study	Country	KT capacity building activity/level	Evaluation method	Target audience
Integrated efforts	Ekirapa-Kiracho et al 2014: Evaluation of a health systems knowledge translation network for Africa (KTNET): a study protocol	Rwanda, Senegal, South Africa, Burundi, the Democratic Republic of Congo, Ghana, Uganda, Ethiopia	Technical Assistance<br>-<br>Systems	Pre/Post Intervention Questionnaire, Interviews, Usage Analytics, Social Network Analysis	8 participating research coalitions
Integrated efforts	El-Jardali et al 2014: Capturing lessons learned from evidence-to-policy initiatives through structured reflection	Argentina, Bangladesh, Nigeria, Burkina Faso, Cameroon, Central African Republic, Ethiopia, Uganda, Sudan, Zambia	KTP<br>-<br>Systems	Interviews, Document review, Observations	KT platform leaders, policymakers, stakeholders
Integrated efforts	Johnson et al 2010: Procedures Manual for Evaluating Knowledge-Translation Platforms in Low- and Middle- Income Countries	Canada	KTP<br>-<br>Systems	Case Study (surveys, media analysis, document review, usage analytics)	policymakers, stakeholders, researchers, participating KTPs & affiliates
Integrated efforts	Kothari et al 2014: Evaluation of partnerships in a transnational family violence prevention network using an integrated knowledge translation and exchange model: a mixed methods study	Canada, the United States of America, Asia, Europe, Australia	Network<br>-<br>Systems	Questionnaire, Interviews	researchers & knowledge users, trainees
Integrated efforts	Mavoa et al 2012: Knowledge exchange in the Pacific: The TROPIC (Translational Research into Obesity Prevention Policies for Communities) project	Fiji	Knowledge broker<br>-<br>Organizational	Interviews, questionnaire, process diaries,	Researchers, policymakers, policymaking organizations, advocacy groups
Integrated efforts	Ongolo-Zogo et al 2014: Initiatives supporting evidence informed health system policymaking in Cameroon and Uganda: a comparative historical case study	Cameroon, Uganda	KTP<br>-<br>Systems	Case Study (document review & questionnaire)	policymakers, researchers, civil society, media

KT Model	Study	Country	KT capacity building activity/level	Evaluation method	Target audience
Integrated efforts	Waqa et al 2013: Participants' perceptions of a knowledge-brokering strategy to facilitate evidence-informed policy-making in Fiji	Fiji	Knowledge broker<br>-<br>Organizational	feedback questionnaire and interviews	academic institutions, research centers, policymaking experts, government departments, NGOs
Integrated efforts	Waqa et al 2013: Knowledge brokering between researchers and policymakers in Fiji to develop policies to reduce obesity: a process evaluation	Fiji	Knowledge broker<br>-<br>Organizational	feedback questionnaire and interviews	academic institutions, research centers, policymaking experts, government departments, NGOs
