# Supplementary material for: Indicators to evaluate organisational knowledge brokers: a scoping review
Source: Health Res Policy Syst. 2020 Aug 24;18:93. doi: 10.1186/s12961-020-00607-8 (PMC7444249; doi:10.1186/s12961-020-00607-8)
Supplement: Supplementary file 3 — Additional file 3. Indicator data extracted from eligible studies to inform their use in evaluation. This file has a complete list of output and outcome indicators based on methods beyond expert opinion that were extracted from the eligible studies. The table includes the indicators organised by domain, the modes of verification for the indicators, the method the indicators were based on or collected with, the frequency of collection, and the study the indicator was extracted from. [file 12961_2020_607_MOESM3_ESM.docx]

| **Domain 1: General climate** | | | | | |
| --- | --- | --- | --- | --- | --- |
| **Indicators** | **Type**  **(quantitative/ qualitative)** | **Mode of verification** | **Method** | **Frequency** | **Literature source** |
| 1.1. # activities identified | qn. | interviews, observations at international forum, document review (forum report) | based on the Lavis assessing country level efforts framework | none explicitly stated; one-time program evaluation | El-Jardali et al 2014 |
| 1.2. Availability of synthesized and packaged evidence | qn./ql. | platform survey responses, usage analytics | field tested but unclear where indicators came from | annual profile collected at baseline and at three consecutive follow up years; outcomes evaluation at baseline, 1.5-year mark, and 3-year mark; case study at 3^rd^ year of intervention; focus group in 4^th^ year of intervention | Johnson et al 2010 |
| 1.3. The organization has the skills, structures, processes and a culture to promote and use research findings in decision-making* | qn./ql. | Is Research Working for You? tool | uses validated assessment tool Is research working for you | twice (pre/post capacity measures) | Mavoa et al 2012 Waqa et al 2013 (2 studies) |
| 1.4. Feedback on context/culture | ql. | phone interviews, email survey, email discussion, webcast | framework, tested in case study | none explicitly stated: one-time post program evaluation | Conklin et al 2008 |
| 1.5. Facilitators, barriers, lessons learnt | ql. | interviews, observations at international forum, document review (forum report) | based on the Lavis assessing country level efforts framework | none explicitly stated; one-time program evaluation | El-Jardali et al 2014 |
| 1.6. Increased demand or value of KT products or knowledge from policy-makers | ql. | feedback from policymakers, interviews, observations at international forum, document review | realist evaluation using independent evaluator  framework | none explicitly stated; one-time program evaluation | Langlois et al 2016 El-Jardali et al 2014 |
| 1.7. # of times evidence is mentioned in policy/parliamentary discussions | qn. | Policy documents | developed with interviews and refined with focus group judged to be credible, feasible, clear, and relevant | none stated: the article developed the indicators, but did not use them to evaluate in this study | Kothari et al 2011 |
| 1.8. Increased awareness of importance of EIP initiatives | ql. | interviews, observations at international forum, document review (forum report) | framework | none explicitly stated: one-time post program evaluation | El-Jardali et al 2014 |
| 1.9. Changes in government allocated funding | qn./ql. | Interviews | non-systematic lit review, expert opinion (interviews), review framework | none explicitly stated; one-time program evaluation | Cole et al 2016 |
| **Domain 2: Production of research** | | | | | |
| **Indicators** | **Type**  **(quantitative/ qualitative)** | **Mode of verification** | **Method** | **Frequency** | **Literature source** |
| 2.1. # peer reviewed journal articles | qn. | PI curricular, Google Scholar database; Publications; Citation index, measurement of research output (annual reports, M&E reports, annual external reviews), MOH information systems, questionnaire | conceptual framework guided evaluation  framework | Twice (pre and post questionnaire); annually | Ekirapa-Kiracho et al 2014 Angulo-Tuesta et al 2016 Kwan et al 2007 |
| 2.2. Citations per article | qn. |  |  |  |  |
| 2.3. Citation of research results by other researchers | ql. |  |  |  |  |
| 2.4. Journal impact factor | qn. | PI publication counts and h index, MOH information system, citation review, questionnaire | framework | none explicitly stated; one-time program evaluation | Angulo-Tuesta et al 2016 Kwan et al 2007 |
| 2.5. # of projects per research approach | qn. | PI publication counts and h index, MOH information system, citation review, questionnaire | framework | none explicitly stated; one-time program evaluation | Angulo-Tuesta et al 2016 Kwan et al 2007 |
| 2.6. Funds invested per project | qn. | PI publication counts and h index, MOH information system, citation review, questionnaire | framework | none explicitly stated; one-time program evaluation | Angulo-Tuesta et al 2016 Kwan et al 2007 |
| 2.7. Project duration | qn. | questionnaire based on the payback framework | payback evaluation framework questionnaire developed by the Health Economics Research Group at Brunel University, UK | one-time data collection | Kwan et al 2007 |
| 2.8. # projects liaising with users | qn. | questionnaire based on the payback framework | payback evaluation framework questionnaire developed by the Health Economics Research Group at Brunel University, UK | one-time data collection | Kwan et al 2007 |
| 2.9. # of projects that led to subsequent research | qn. |  |  |  |  |
| 2.10. Researcher feedback on project alignment with priorities | ql. | website material, document review (proposals, reports on the national research agenda, annual monitoring and evaluation reports, prior independent evaluations, and programs of dissemination conferences), interviews, team data files of all project topics and awardees. | non-systematic lit review, expert opinion (interviews), review framework | none explicitly stated; one-time program evaluation | Cole et al 2016 |
| 2.11. Mean score of scientific accuracy | qn. | Written questionnaire, PI publication counts and h index, MOH information system, citation review | framework | none explicitly stated; one-time program evaluation | Angulo-Tuesta et al 2016 |
| 2.12. Mean score of readability | qn. |  |  |  |  |
| 2.13. Mean score of usability | qn. |  |  |  |  |
| 2.14. Mean score of ease of access | qn. |  |  |  |  |
| 2.15. Applicability of research for decision making | ql. |  |  |  |  |
| 2.16. Developed priority report (i.e. research agenda, list of priorities, country assessment) | ql. | Document review, material available on websites, annual monitoring and evaluation reports | non-systematic lit review, expert opinion (interviews), review framework | none explicitly stated; one-time program evaluation | Cole et al 2016 |
| 2.17. Revision with stakeholders | ql. |  |  |  |  |
| 2.18. Feedback on support and/or awareness | ql. | Interviews | non-systematic lit review, expert opinion (interviews), review framework | none explicitly stated; one-time program evaluation | Cole et al 2016 |
| 2.19. Feedback on priority development | ql. |  |  |  |  |
| 2.20. Feedback on priorities | ql. |  |  |  |  |
| 2.21. Changes in policies or programs consistent with evidence produced | ql. | databases (administrative records), interviews, surveys, focus groups, direct observation, document review, chart audits | framework | some data collected annually (annual reports)  other data collected as part of the evaluation review (interview data) | Alberta health services 2014 |
| 2.22. Policy-makers, stakeholders, and researchers report that relevant and understandable health research evidence is more readily available and cite this research evidence in media | ql. | platform survey responses, usage analytics; annual profile; case study; focus groups | field tested but unclear where indicators came from | annual profile collected at baseline and at three consecutive follow up years; outcomes evaluation at baseline, 1.5-year mark, and 3-year mark; case study at 3^rd^ year of intervention; focus group in 4^th^ year of intervention | Johnson et al 2010 |
| **Domain 3: KT activities: push efforts** | | | | | |
| **Indicators** | **Type**  **(quantitative/ qualitative)** | **Mode of verification** | **Method** | **Frequency** | **Literature source** |
| 3.1. # of downloads | qn. | website tracking, survey, telephone interview | conceptual framework guided evaluation | pre/post or quarterly | Ekirapa-Kiracho et al 2014 |
| 3.2. # of page visits (total and unique) | qn. |  |  |  |  |
| 3.3. # of countries visiting the website | qn. |  |  |  |  |
| 3.4. # of page views per visit | qn. |  |  |  |  |
| 3.5. # of requests for materials | qn. | databases (administrative records), interviews, surveys, focus groups, direct observation, document review, chart audits | adapted from USAID evaluation guides | stated that frequency should be determined as part of the evaluation planning process | Alberta health services 2014 |
| 3.6. Extent of media exposure | ql. |  |  |  |  |
| 3.7. Referrals made to distributed materials | ql. |  |  |  |  |
| 3.8. # of materials distributed | qn. |  |  |  |  |
| 3.9. Transmitted to relevant stakeholder (discussed at policy dialogues, dissemination workshops) | ql. | Online/telephone survey | conceptual framework guided evaluation | quarterly | Ekirapa-Kiracho et al 2014 |
| 3.10. Disseminated materials are read and understood | ql. |  |  |  |  |
| 3.11. Efforts have been made to adopt the disseminated knowledge | ql. |  |  |  |  |
| 3.12. Platform survey responses | ql. |  |  |  |  |
| 3.13. Usage analytics of promotional products | qn. |  |  |  |  |
| 3.14. Research is presented to decision makers in a useful way* | qn./ql. | Is Research Working for You? tool | uses validated assessment tool Is research working for you | twice (pre/post capacity measures) | Mavoa et al 2012  Waqa et al 2013 (2 studies) |
| 3.15. Multiple formats of written and/or other forms of presentation (e.g. newsletter, website summary, interim report, oral presentation) | ql. | qualitative interviews and focus group | developed with interviews and refined with focus group judged to be credible, feasible, clear, and relevant | none stated: the article developed the indicators, but did not use them to evaluate in this study | Kothari et al 2011 Kothari et al 2014 |
| 3.16. Presentation formats include layman’s terms and recommendations | ql. |  |  |  |  |
| 3.17. Where appropriate, presentation formats are concise (e.g. less than two pages) | ql. |  |  |  |  |
| 3.18. Users contacted researchers to discuss results | ql. |  |  |  |  |
| 3.19. Relevant documents disseminated in hardcopy | ql. | interviews | none stated | none stated | The CIPHER Investigators 2014 |
| 3.20. Website or online evidence database is established | ql. | annual profile/inventory; case study; focus groups | field tested but unclear where indicators came from | annual profile collected at baseline and at three consecutive follow up years; outcomes evaluation at baseline, 1.5 year mark, and 3 year mark; case study at 3^rd^ year of intervention; focus group in 4^th^ year of intervention | Johnson et al 2010 |
| 3.21. # of dissemination workshops | qn. | none stated | not systematically for M&E; partly used Maselli, Lys and Schmid (2005) criteria  also used expert opinion conceptual framework | none explicitly stated; one-time program evaluation | Aikins et al 2012 |
| 3.22. % of grantees presenting at conferences | qn. | Interviews | non-systematic lit review, expert opinion (interviews), review framework | none explicitly stated; one-time program evaluation | Cole et al 2016 |
| 3.23. % of grantees submitting work for publication | qn. |  |  |  |  |
| 3.24. % of grantees with published research at time of review | qn. |  |  |  |  |
| 3.25. Feedback from grantees on competence & opportunities for dissemination | ql. |  |  |  |  |
| 3.26. #/amount of grant (applications) | qn. | website material, document review, interviews, team data files of all project topics and awardees, Data files on projects/awardees; funder information systems; document review | non-systematic review, expert opinion (interviews), review framework; questionnaire developed for study, analytic framework | none explicitly stated; one-time program evaluation | Cole et al 2016 Ongolo-Zogo et al 2014 Aikins et al 2012 |
| 3.27. # of researcher internships | qn. | none stated | not systematically for M&E; partly used Maselli, Lys and Schmid (2005) criteria  also used expert opinion conceptual framework | none explicitly stated; one-time program evaluation | Aikins et al 2012 |
| 3.28. # of trainees publishing research | qn. | Data files on projects/awardees; annual M&E reports; Publications; Citation index | not systematically for M&E; partly used Maselli, Lys and Schmid (2005) criteria  also used expert opinion conceptual framework | none explicitly stated; one-time program evaluation | Aikins et al 2012 |
| 3.29. Feedback on improved quality of research results | ql. | website material, document review, interviews, team data files of all project topics and awardees. | non-systematic lit review, expert opinion (interviews), review framework | none explicitly stated; one-time program evaluation | Cole et al 2016 |
| 3.30. % of research applications headed by a national | qn. |  |  |  |  |
| 3.31. Increased interest by young nationals in research | qn./ql. |  |  |  |  |
| 3.32. Establishment of a PhD program | ql. | website, strategic plans, annual reports | questionnaire developed for study, analytic framework, | none explicitly stated; one-time program evaluation | Ongolo-Zogo et al 2014 |
| 3.33. # of projects supported | qn. | website material, document review, interviews, team data files of all project topics and awardees. | non-systematic lit review, expert opinion (interviews), review framework | none explicitly stated; one-time program evaluation | Cole et al 2016 |
| 3.34. # of project findings used/expected to be used in policy | qn. | questionnaire based on the payback framework | framework | one-time data collection | Kwan et al 2007 |
| 3.35. # of projects leading to/expecting to change behavior | qn. | questionnaire based on the payback framework | framework | one-time data collection | Kwan et al 2007 |
| 3.36. Increase in inquiries and applications | qn. | website material, document review (proposals, reports on the national research agenda, annual monitoring and evaluation reports, prior independent evaluations, and programs of dissemination conferences), interviews, team data files of all project topics and awardees. | non-systematic lit review, expert opinion (interviews), review framework | none explicitly stated; one-time program evaluation | Cole et al 2016 |
| 3.37. Phasing out of external funding | ql. | Interviews | non-systematic lit review, expert opinion (interviews), review framework | none explicitly stated; one-time program evaluation | Cole et al 2016 |
| **Domain 4: KT activities: pull efforts** | | | | | |
| **Indicators** | **Type**  **(quantitative/ qualitative)** | **Mode of verification** | **Method** | **Frequency** | **Literature source** |
| 4.1. Seeking, Engaging with, and Evaluating Research (SEER)* | qn./ql. | SEER measurement tool: online survey | All indicators were developed for the trial since they couldn't find quality ones in literature. validated, piloted | Outcomes collected every 6 months for 30 months (trial was only one year) | The CIPHER Investigators 2014 |
| 4.2. Organizational Research Access, Culture and Leadership (ORACLe)* | qn./ql. | Face-to-face/phone interviews; document review; scores by expert panel | All indicators were developed for the trial since they couldn't find quality ones in literature. validated, piloted | Outcomes collected every 6 months for 30 months (trial was only one year) | The CIPHER Investigators 2014 |
| 4.3. Staff Assessment of enGagement with Evidence (SAGE)* | qn./ql. | SAGE measurement tool: Face-to-face/phone interviews; document review; scores by expert panel | All indicators were developed for the trial since they couldn't find quality ones in literature. validated, piloted | Outcomes collected every 6 months for 30 months (trial was only one year) | The CIPHER Investigators 2014 |
| **Domain 5: KT activities: exchange efforts** | | | | | |
| **Indicators** | **Type**  **(quantitative/ qualitative)** | **Mode of verification** | **Method** | **Frequency** | **Literature source** |
| 5.1. Grants for collaboration | ql. | document review (proposals, reports) annual monitoring and evaluation reports, prior independent evaluations, team data files of all project topics and awardees. | non-systematic lit review, expert opinion (interviews), review framework | none explicitly stated; one-time program evaluation | Cole et al 2016 |
| 5.2. Research projects are produced with policy-makers | ql. | annual profile/inventory, bibliographic database | field tested but unclear where indicators came from | annual profile collected at baseline and at three consecutive follow up years; outcomes evaluation at baseline, 1.5-year mark, and 3-year mark; case study at 3^rd^ year of intervention; focus group in 4^th^ year | Johnson et al 2010 Aikins et al 2012 |
| 5.3. Disciplinary backgrounds of contributing authors | ql. |  |  |  |  |
| 5.4. Invitations to publish special issues | ql. |  |  |  |  |
| 5.5. Partners views on using research results | ql. | interviews, survey, focus group discussion | conceptual framework guided evaluation | Twice (pre/post) | Ekirapa-Kiracho et al 2014 |
| 5.6. Negotiation occurs during the research process | ql. | qualitative interviews and focus group | developed with interviews and refined with focus group judged to be credible, feasible, clear, and relevant | none stated: the article developed the indicators, but did not use them to evaluate in this study | Kothari et al 2011 Kothari et al 2014 |
| 5.7. Negotiated items are clearly understood by all | ql. |  |  |  |  |
| 5.8. Deciding on objectives together | ql. | none stated | Maselli, Lys and Schmid (2005) framework | none explicitly stated; one-time program evaluation | Aikins et al 2012 |
| 5.9. Built mutual trust | ql. |  |  |  |  |
| 5.10. Communication tools established | ql. |  |  |  |  |
| 5.11. Sharing of information and responsibility | ql. |  |  |  |  |
| 5.12. Transparency | ql. |  |  |  |  |
| 5.13. Share profits equally | ql. |  |  |  |  |
| 5.14. Build on achievements | ql. |  |  |  |  |
| 5.15. Communication is clear | ql. | qualitative interviews and focus group | developed with interviews and refined with focus group judged to be credible, feasible, clear, and relevant | none stated: the article developed the indicators, but did not use them to evaluate in this study | Kothari et al 2011 Kothari et al 2014 |
| 5.16. Communication is relevant | ql. |  |  |  |  |
| 5.17. Communication is timely | ql. |  |  |  |  |
| 5.18. Communication is respectful | ql. |  |  |  |  |
| 5.19. Density and centrality | qn. | Social network analysis | conceptual framework guided evaluation | quarterly | Ekirapa-Kiracho et al 2014 |
| 5.20. Connectedness of networks | ql. |  |  |  |  |
| 5.21. Partners mention each other | ql. |  |  |  |  |
| 5.22. Partners are flexible about meeting partner’s changing needs and revising research plans and timelines | ql. | qualitative interviews and focus group | developed with interviews and refined with focus group judged to be credible, feasible, clear, and relevant | none stated: the article developed the indicators, but did not use them to evaluate in this study | Kothari et al 2011 Kothari et al 2014 |
| 5.23. Partners understand the limits of each other’s flexibility | ql. |  |  |  |  |
| 5.24. Partners understand research findings, their limits, and their implications for Ministry work | ql. |  |  |  |  |
| 5.25. Conflict is dealt with openly, informally, and promptly | ql. |  |  |  |  |
| 5.26. Trust has increased between partners | ql. |  |  |  |  |
| 5.27. Comfort has increased between partners | ql. |  |  |  |  |
| 5.28. Openness has increased between partners | ql. |  |  |  |  |
| 5.29. Partners begin speaking a common language regarding research | ql. |  |  |  |  |
| 5.30. Partners facilitate removal of barriers for each other’s work | ql. |  |  |  |  |
| 5.31. Linkage with partner enhances partner linkage with community/other stakeholders | ql. |  |  |  |  |
| 5.32. There is joint commitment to the research project | ql. |  |  |  |  |
| 5.33. There is an increase in joint activity around the project | ql. |  |  |  |  |
| 5.34. Clear leadership of partnerships | ql. |  |  |  |  |
| 5.35. Team mentality | ql. |  |  |  |  |
| 5.36. Early engagement of members | ql. |  |  |  |  |
| 5.37. # of members | qn. | none stated | expert opinion | none explicitly stated; one-time program evaluation | Aikins et al 2012 |
| 5.38. #/% of members present at activities | qn. |  |  |  |  |
| 5.39. Level of engagement | qn./ql. |  |  |  |  |
| 5.40. #/% partners active | qn. |  |  |  |  |
| 5.41. Member affiliation and profession | ql. |  |  |  |  |
| 5.42. Joint meetings occur at most stages of research | ql. | qualitative interviews and focus group | developed with interviews and refined with focus group judged to be credible, feasible, clear, and relevant | none stated: the article developed the indicators, but did not use them to evaluate in this study | Kothari et al 2011 Kothari et al 2014 |
| 5.43. Joint meetings occur to discuss research dissemination and utilization plans | ql. |  |  |  |  |
| 5.44 Feedback on linkage and exchange mechanisms | ql. | phone interviews, email survey, email discussion, webcast | framework, tested in case study | none explicitly stated: one-time post program evaluation | Conklin et al 2008 |
| 5.45. # partners involved in KT activities | qn. | key informant interviews, social network analysis, online/telephone survey, focus group discussion | conceptual framework guided evaluation | Twice (pre and post questionnaire) | Ekirapa-Kiracho et al 2014 |
| 5.46. Stakeholders involved | ql. | Document review, messages between researchers on an online forum, policy documents, technical/evidence inputs from the researcher buddies, and news media), | realist evaluation using independent evaluator | none explicitly stated; one-time program evaluation | Langlois et al 2016 |
| 5.47. Partners are perceived as experts in the research/policy area and are referred to as such to others | ql. | qualitative interviews and focus group | developed with interviews and refined with focus group judged to be credible, feasible, clear, and relevant | none stated: the article developed the indicators, but did not use them to evaluate in this study | Kothari et al 2011 Kothari et al 2014 |
| 5.48. Value of network | ql. |  |  |  |  |
| 5.49. Feedback on awareness and perceptions of network | ql. | phone interviews, email survey, email discussion, webcast | framework, tested in case study | none explicitly stated: one-time post program evaluation | Conklin et al 2008 |
| 5.50. Partnerships are built and sustained | ql. | Case study using interviews, focus groups, field notes, online survey, document review | case study framework; team members input | none explicitly stated: one-time program evaluation | Murnaghan et al 2013 |
| **Domain 6: KT activities: integrated efforts** | | | | | |
| **Indicators** | **Type**  **(quantitative/ qualitative)** | **Mode of verification** | **Method** | **Frequency** | **Literature source** |
| 6.1. # of KTPs viewing their work as a long-term initiative | qn. | interviews, observations at international forum, document review (forum report) | Framework | none explicitly stated; one-time program evaluation | El-Jardali et al 2014 |
| 6.2. # of KTPs engaging in priority setting with stakeholders | qn. | Individual/group face-to-face interviews, deliberation observations, forum reports | Framework | none explicitly stated; one-time program evaluation | El-Jardali et al 2014 |
| 6.3. # of KTPs building capacity for priority setting | qn. | Individual/group face-to-face interviews, deliberation observations, forum reports | Framework | none explicitly stated; one-time program evaluation | El-Jardali et al 2014 |
| 6.4. # of KTPs producing/in process of KT products (by type; e.g. evidence briefs, clearinghouses, rapid response services, deliberative dialogues, systematic reviews) | qn. | Annual profile/inventory; Individual/group face-to-face interviews, deliberation observations, forum reports | Framework | none explicitly stated; one-time program evaluation | El-Jardali et al 2014 |
| 6.5. # KTPs that built capacity for KT (evidence briefs; deliberative dialogues; accessing, assessing, and using research evidence) | qn. | Individual/group face-to-face interviews, deliberation observations, forum reports | Framework | none explicitly stated; one-time program evaluation | El-Jardali et al. 2014 |
| 6.6. # KTPs training research users in KT (systematic reviews, evidence briefs, deliberative dialogues) | qn. | Individual/group face-to-face interviews, deliberation observations, forum reports | Framework | none explicitly stated; one-time program evaluation | El-Jardali et al. 2014 |
| 6.7. # of organizations using the products | qn. | interviews, process diaries | none stated | none stated | Waqa et al 2013 (2 studies) |
| 6.8. Functional website or clearinghouse providing KT resources | ql. | Annual profile/inventory; website material | questionnaire developed for study, analytic framework, | none explicitly stated; one-time program evaluation | Ongolo-Zogo et al 2014 |
| 6.9. Amount of resources utilized in knowledge brokering activities (e.g. cost, time, materials) | qn./ql. | Process diaries; meeting minutes; emails; phone calls; interactions | none stated | none stated | Mavoa et al 2012 Waqa et al 2013 (2 studies) |
| 6.10. # of KT materials | qn. | website, evaluation survey, grant/annual reports, pre/post questionnaire | questionnaire developed for study, analytic framework,   framework guided evaluation | none explicitly stated; one-time program evaluation | Ongolo-Zogo et al 2014 Ekirapa-Kiracho et al 2014 |
| 6.11. Products (e.g. website, policy dialogues, evidence briefs) aligned with and address priorities | ql. | Evaluation survey | questionnaire developed for study, analytic framework, | none explicitly stated; one-time program evaluation | Ongolo-Zogo et al 2014 |
| 6.12. Topic of KT materials | ql. | website, evaluation survey, grant/annual reports, pre/post questionnaire | questionnaire developed for study, analytic framework,   framework guided evaluation | none explicitly stated; one-time program evaluation | Ongolo-Zogo et al 2014 Ekirapa-Kiracho et al 2014 |
| 6.13. # of KT materials translated/available in different languages | qn. |  |  |  |  |
| 6.14. Policy dialogues about high-priority policy issues take place regularly | ql. | Annual profile/inventory; case study; focus groups | field tested but unclear where indicators came from | annual profile collected at baseline and at three consecutive follow up years; outcomes evaluation at baseline, 1.5 year mark, and 3 year mark; case study at 3^rd^ year of intervention; focus group in 4^th^ year of intervention | Johnson et al 2010 |
| 6.15. Scoring of quality dimensions (Mean, Standard deviation) | qn. | Evaluation survey, pre/post questionnaire | questionnaire developed for study, analytic framework,   framework guided evaluation | none explicitly stated; one-time program evaluation;  pre/post intervention | Ongolo-Zogo et al 2014 Ekirapa-Kiracho et al 2014 |
| 6.16. KT activities regarded as beneficial for bringing together stakeholders and facilitating the development of partnerships. | ql. | semi structured interviews, focus groups, field notes, online survey, planning and resource documents, meeting minutes, grant applications, communications and press clippings | -case study framework -team members input | none explicitly stated: one-time program evaluation using case study | Murnaghan et al 2013 |
| 6.17. Uptake and/or influence of evidence (reports, policy briefs, recommendations, other) in decision making | ql. | document review and analysis, interviews, quantitative surveys website tracking, success stories, social network analysis, online/telephone survey, focus group discussion, citation review, media clippings, observation, chart audits | Frameworks | capacity was measured twice (pre/post)  quarterly  none explicitly stated; one-time program evaluation | Ekirapa-Kiracho et al 2014 Angulo-Tuesta et al 2016 Murnaghan et al 2013 Alberta health services 2014 |
| 6.18. Financial & organizational support to the KTP | qn./ql. | website material, document review (proposals, reports on the national research agenda, annual monitoring and evaluation reports, prior independent evaluations, and programs of dissemination conferences), interviews, team data files of all project topics and awardees. | non-systematic lit review, expert opinion (interviews), review framework | none explicitly stated; one-time program evaluation | Cole et al 2016 |
| **Domain 7: Evaluation** | | | | | |
| **Indicators** | **Type**  **(quantitative/ qualitative)** | **Mode of verification** | **Method** | **Frequency** | **Literature source** |
| 7.1. # of KTPs evaluating KT product(s) quality | qn. | Individual/group face-to-face interviews, deliberation observations, forum reports | Framework | none explicitly stated; one-time program evaluation | El-Jardali et al 2014 |
| 7.2. Perception of sustainability | ql. | interviews, observations at international forum, document review (forum report) | Framework | none explicitly stated; one-time program evaluation | El-Jardali et al 2014 |
| **Domain 8: Capacity building** | | | | | |
| **Indicators** | **Type**  **(quantitative/ qualitative)** | **Mode of verification** | **Method** | **Frequency** | **Literature source** |
| 8.1. # of activities | qn. | Annual profile/inventory, evaluation survey | questionnaire developed for study, analytic framework | one-time program evaluation | Ongolo-Zogo et al 2014 |
| 8.2. type of activity | ql. |  |  |  |  |
| 8.3. # of people invited | qn. | assessment questionnaire; activity reports; Certificates; internal data reporting; annual profile | scoring based on Johnson McMaster methods (MEAN, Range); framework; questionnaire developed for study; analytic framework | Twice (pre-workshop and post workshop)  one-time program evaluation | Uneke et al 2015a Uneke et al 2015b Uneke et al 2011 Angulo-Tuesta et al 2016 Ongolo-Zogo et al 2014 Waqa et al 2013 (2 studies) Neves et al 2014 Aikins et al 2012 |
| 8.4. # of people attended | qn. |  |  |  |  |
| 8.5. # people trained | qn. |  |  |  |  |
| 8.6. Reasons for participation non completion | ql. |  |  |  |  |
| 8.7. Participant occupation | ql. | pre/post survey, event reports, questionnaire | scoring  analysis based on Johnson McMaster methods (MEAN, Range)  framework/unclear | Twice (pre-workshop and post workshop) or post workshop only | Uneke et al 2015a Uneke et al 2015b Uneke et al 2011 Ongolo-Zogo et al 2014 Waqa et al 2013 (2 studies)  Neves et al 2014 |
| 8.8. Participant affiliation | ql. |  |  |  |  |
| 8.9. Participant education level | ql. |  |  |  |  |
| 8.10. Participant gender | ql. |  |  |  |  |
| 8.11. Participant age | qn. |  |  |  |  |
| 8.12. Participant's # of years in current position | qn. |  |  |  |  |
| 8.13. Participant's level of policy influence | ql. |  |  |  |  |
| 8.14. Country of participants | ql. |  |  |  |  |
| 8.15. Participant's years of experience with evidence-informed policy-making | qn. |  |  |  |  |
| 8.16. Training workshops for policy-makers and researchers are designed and implemented regularly | ql. | Annual profile/inventory; case study; focus groups | field tested but unclear where indicators came from | annual profile collected at baseline and at three consecutive follow up years; outcomes evaluation at baseline, 1.5 year mark, and 3 year mark; case study at 3^rd^ year of intervention; focus group in 4^th^ year of intervention | Johnson et al 2010 |
| 8.17. Mean program ratings & feedback | qn. | Assessment questionnaire, | scoring  analysis based on Johnson McMaster methods (MEAN, Range); previously published conference evaluation survey and input from meeting organizers and pre-determined objectives | Twice (pre-workshop and post workshop)  Post intervention | Uneke et al 2015a  Uneke et al 2015b  Uneke et al 2011 Neves et al 2014 |
| 8.18. Intent to return | ql. |  |  |  |  |
| 8.19. Survey response rate | qn. | Assessment questionnaire | previously published conference evaluation survey and input from meeting organizers and pre-determined objectives | post workshop (once only) | Neves et al 2014 |
| 8.20. % increase in pre/post scores of skill abilities (e.g. access evidence, synthesize evidence, policy dialogues, evidence briefs, collaboration etc.) and value of knowledge use | qn. | pre/post survey; annual reports | scoring  analysis based on Johnson McMaster methods (MEAN, Range) | Twice (pre-workshop and post workshop) | Uneke et al 2015a  Uneke et al 2015b  Uneke et al 2011 Johnson et al |
| 8.21. Comments in the media reflect capacity changes | ql. |  |  |  |  |
| 8.22. Ability to acquire research | qn. |  |  |  |  |
| 8.23. Increased research capacity | qn. |  |  |  |  |
| 8.24. Change in research/policy-maker relationship | ql. | document review and analysis, interviews, quantitative surveys, observations, | Framework | none stated | El-Jardali et al 2014 Johnson et al |
| 8.25. Comments in the media reflect relationship changes | ql. |  |  |  |  |
| 8.26. Perceived EIP skills/changes in skills (acquire, assess, adapt, apply)* | qn./ql. | semi-structured interviews,  self-assessment tool (Is Research Working For You?) process diary (intervention activities) | uses validated assessment tool Is research working for you | twice (pre/post capacity measures) | Mavoa et al 2012 Waqa et al 2013 (2 studies) |
| 8.27. # participants reporting benefits | qn. | Assessment questionnaire | based on previously published conference evaluation survey, input from meeting organizers, and pre-determined objectives | post workshop (once only) | Neves et al 2014 |
| 8.28. Awareness of key government documents | qn./ql. | semi-structured interviews | unclear | post intervention | Waqa et al 2013 (2 studies) |
| 8.29. Perceived change in skills & confidence to interact with experts | ql. | project report, project meeting minutes and emails, messages between researchers on an online forum | realist evaluation using independent evaluator | none explicitly stated; one-time program evaluation | Langlois et al 2016 |
| 8.30. Perceived impact on current position and/or future career advancement* | qn./ql. | Interviews; assessment questionnaire, Is Research working for you? measurement tool | uses validated assessment tool Is research working for you | twice (pre/post capacity measures) | Mavoa et al 2012 Kwan et al 2007 |
| 8.31. Contribution to decision making by partners and policy-makers | ql. | key informant interviews, social network analysis, online/telephone survey, focus group discussion | conceptual framework guided evaluation | Twice (pre and post questionnaire) | Ekirapa-Kiracho et al 2014 |
| 8.32. Feedback on behavioral changes | ql. | phone interviews, email survey, document review, email discussion, webcast | framework, tested in case study | none explicitly stated: one-time post program evaluation | Conklin et al 2008 |
| 8.33. #/% of trainees reporting intent to use skills gained | qn. | post intervention questionnaire | based on previously published conference evaluation survey, input from meeting organizers and pre-determined objectives | post workshop (once only) | Neves et al 2014 |
